# Impact on parasitemia, survival time and pro-inflammatory immune response in mice infected with *Plasmodium berghei* treated with *Eleutherine plicata*

**DOI:** 10.3389/fphar.2024.1484934

**Published:** 2024-12-05

**Authors:** Antônio Rafael Quadros Gomes, Ana Laura Gadelha Castro, Gleison Gonçalves Ferreira, Heliton Patrick Cordovil Brígido, Everton Luiz Pompeu Varela, Valdicley Vieira Vale, Liliane Almeida Carneiro, Maria Fâni Dolabela, Sandro Percario

**Affiliations:** ^1^ Postgraduate Program in Pharmaceutical Innovation, Federal University of Pará, Belém, Brazil; ^2^ Postgraduate Program in Biodiversity and Biotechnology, Federal University of Pará, Belém, Brazil; ^3^ Postgraduate Program in Pharmaceutical Sciences, Federal University of Pará, Belém, Brazil; ^4^ Oxidative Stress Laboratory, Institute of Biological Sciences, Federal University of Pará, Belém, Brazil; ^5^ National Primate Center, Instituto Evandro Chagas, Ananindeua, Brazil

**Keywords:** eleutherol, eleutherol glucuronide, isoeleutherin, eleutherin, malaria

## Abstract

*In vitro* studies with *Plasmodium falciparum* have demonstrated the antiparasitic activity of *E. plicata*, attributed to its naphthoquinones. This study reports on pro-inflammatory changes in mice infected with *P. berghei* and correlates these changes with parasitemia and survival. The ethanol extract of *Eleutherine plicata* (EEEp) was fractionated under reflux to obtain the dichloromethane fraction (FDMEp) and isolated compounds from *E. plicata*, relating these to survival time and parasitemia. Antimalarial activity was evaluated using the Peters suppressive test, with mice infected with *Plasmodium berghei* and treated with *E. plicata*, assessing parasitemia and survival over 30 days. The pro-inflammatory profile was determined by measuring interleukin-10, interferon-γ (IFN-γ), and nitric oxide levels. EEEp, FDMEp, and eleutherol showed activity on the 5th day of infection, with only FDMEp being active on the 8th day. Treatment with EEEp and FDMEp extended animal survival, reduced IFN-γ and NO levels, and increased IL-10 levels. Eleutherol significantly altered the response, with eleutherol glucuronide seemingly active by binding to lactate dehydrogenase, inhibiting hemozoin metabolism, leading to parasite death. Pro-inflammatory changes did not appear to correlate with survival and reduced parasitemia. In summary, FDMEp and eleutherol reduced parasitemia, extended survival, and modulated the inflammatory response. FDMEp and eleutherol are promising candidates for developing new antimalarial drugs.

## 1 Introduction

Malaria is one of the most prevalent human infections globally and a significant health problem. In 2022, an estimated 249 million cases of the disease were reported worldwide, surpassing the pre-pandemic level of 233 million in 2019. In Latin America, approximately 138 million people are at risk of contracting malaria, with 520,000 cases reported in 2021. Venezuela, Brazil, and Colombia together accounted for 80.0% of the autochthonous cases on the South American continent (WHO, 2022).

The main *Plasmodium* species are *Plasmodium vivax* and *Plasmodium falciparum*, with infections caused by *P. falciparum* responsible for the most severe forms of the disease and nearly all deaths (Hogg et al., 2006; WHO, 2019). In malaria-endemic regions, repeated infection can lead to antigenic reaction and immunity due to immunoglobulin G (IgG) targeting surface antigens of infected erythrocytes (VSA; Muehlenbachs et al., 2007; Burel et al., 2017), promoting cytoadherence and vascular obstruction (Kraisin et al., 2020).

Cerebral malaria is a fatal complication involving oxidative stress (Akhoon et al., 2014), increasing erythrocyte adhesion in vessels and causing blood flow obstruction, resulting in ischemia-reperfusion syndrome. The release of heme from lysed erythrocytes activates the immune response through the release of pro-inflammatory cytokines, such as interferon-γ (IFN-γ), tumor necrosis factor-α (TNF-α), and lymphotoxin-α by phagocytic cells during respiratory burst. These conditions in lung and brain tissues increase microvascular permeability, resulting in pulmonary syndrome associated with severe malaria (Gomes et al., 2022).

In rodent models, *Plasmodium berghei* infection induces liver injury, increases mRNA expression of interleukin-12 (IL-12), protein 40 (p40), as well as IFN-γ, interleukin-4 (IL-4), and inetrleukin-10 (IL-10), with a consequent increase in nitric oxide (NO) synthesis. Searching for drugs with anti-IL-12 activity may indirectly reduce free radical production, prolong survival, reduce liver damage and weight loss, but without altering parasitemia (Yoshimoto et al., 1998; Percario et al., 2012). Inhibiting IFN-γ and increasing IL-10, an attempt to balance anti-inflammatory and pro-inflammatory cytokines, seem fundamental to controlling parasitemia, symptoms, and severity of malaria (Farrington et al., 2017; Kumar et al., 2019).

In malaria, during the erythrocytic phase, there is intense activation of the immune system, with rapid production of pro-inflammatory cytokines such as IFN-γ by different cells and increased NO to control the infection, directing a type 1 response profile (Th1) (Artavanis-Tsakonas and Riley, 2002; McCall and Sauerwein, 2010; Perez-Mazliah and Langhorne, 2015; Mandala et al., 2021). Conversely, elevated IFN-γ levels and reduced IL-10 are associated with worsening infection (Andrade-Neto et al., 2004; Obeagu, 2024). Therefore, to control the exaggerated pro-inflammatory response, Th1 profile effector cells self-regulate by increasing IL-10 production in malaria (Freitas do Rosario and Langhorne, 2012). IL-10 suppresses antigen presentation and T-cell activation by downregulating class II major histocompatibility complex and co-stimulatory molecules like CD80 and CD86 in macrophages and dendritic cells (Ma et al., 2015; Osii et al., 2020). Thus, when the anti-inflammatory profile properly regulates inflammation, there is a delay in disease progression (Chen et al., 2018; Mandala et al., 2021).

Based on this, studies demonstrated that compounds isolated from *Eleutherine plicata* were able to significantly reduce the inflammatory process through the expression of IFN-γ, promoting the regulation of Th1 cells, which provides the species’ anti-inflammatory activity (Hong et al., 2008; Kamarudin et al., 2021). Furthermore, a study conducted by Hanh et al. (2018) demonstrated that the extract was able to reduce the inflammatory process at a concentration of 1,000 mg/kg, with a reduction in inflammatory cytokines such as TNF-α and IL-6. Regarding the inflammatory process mediated by nitric oxide, both the extract and the isolates demonstrated a great potential for its production, with the efficacy of the extract established in 27.30 μg/mL (IC_50_) and of the isolates isoeleutherin and eleutherin in 7.7 and 11.4 μg/mL (IC_50_), respectively (Han et al., 2008; Hanh et al., 2018; Kamarudin et al., 2021).

In this context, the search for compounds with both antiparasitic activity and the ability to modulate the inflammatory response related to parasite infection is important. *Eleutherine plicata* showed *in vitro* activity against *P. falciparum*, and the fractionation of ethanolic extract of *E. plicata* (EEEp) led to the isolation of active fractions and naphthoquinones (eleutherin and isoeleutherin). Eleutherin and isoeleutherin interacted with highly conserved residues of the binding cavity of the cytochrome bc_*1*_ complex, a protein found in mitochondria (Vale et al., 2020). The cytochrome bc_*1*_ respiratory complex comprises three subunits: Rieske iron-sulfur protein (QcrA), cytochrome B subunit (QcrB), and cytochrome C subunit (QcrC) (Ko and Choi, 2016; Bajeli et al., 2020), with the B subunit of cytochrome bc_*1*_ oxidase involved in energy metabolism, specifically electron transport (Cole, 2016; Bajeli et al., 2020).

To identify the involvement of oxidative stress in antimalarial activity, oxidative changes in mice infected with *P. berghei* and treated with EEEp, FDMEp, naphthoquinones, and eleutherol were evaluated. EEEp (200 mg/kg), dichloromethane fraction of *E. plicata* (FDMEp 50 and 100 mg/kg), isoeleutherin (30 mg/kg), eleutherin (45 mg/kg), and eleutherol (15 and 45 mg/kg) increased the trolox-equivalent antioxidant capacity compared to the positive control. Groups treated with extracts and isolated compounds significantly increased reduced glutatione values compared to the negative control. EEEp, FDMEp, eleutherin, isoeleutherin, and eleutherol reduced oxidative stress (Thiobarbituric Acid Reactive Substances - TBARS) compared to the positive control. Molecular docking demonstrated interactions of eleutherol, eleutherin, and isoeleutherin with antioxidant defense enzymes. Eleutherin and isoeleutherin showed the lowest binding energy for catalase, glutathione reductase, and glutathione peroxidase. In summary, *E. plicata* derivatives stimulated increased antioxidant capacity and reduced oxidative stress in mice infected with *P. berghei* (Gomes et al., 2023).

The results suggest that *E. plicata* has anti-inflammatory potential (Gomes et al., 2023), but its interference in the pro-inflammatory response of mice infected with *P. berghei* remains to be clarified. This study describes for the first time the interference of EEEp, FDMEp, naphthoquinones, and eleutherol in the pro-inflammatory response, as well as their correlation with parasitemia and lifespan. Additionally, the metabolism of eleutherol and naphthoquinones was simulated.

## 2 Materials and methods

### 2.1 Materials

#### 2.1.1 Plant and phytochemical study

In another study by our research group, ethanolic extract, dichloromethane fraction, and three pure substances, isoeleutherin, eleutherin, and eleutherol, were obtained and characterized from the bulbs of *E. plicata*. The obtaining and characterization of the extract and fraction, as well as the identification of compounds, are described in Castro et al. (2021).

#### 2.1.2 Animals and provenance

The project was approved by the Ethics Committee for Animal Use - CEUA/UFPA (no. 7464060618, ID 001020). A total of 195 female mice of the species *Mus musculus*, BALB-c lineage (25–30 g), were used, sourced from the animal facility of the Evandro Chagas Institute (IEC; Ananindeua-PA, Brazil). The animals were housed in cages of five animals each, under controlled conditions of temperature (25°C ± 1°C) with a 12-h light/dark cycle, and had access to water and food *ad libitum*. All procedures involving animals were in accordance with animal experimentation standards, following the ethical principles of experimentation, according to the Brazilian Society of Laboratory Animal Science - SBCAL guidelines.

### 2.2 Detailed methods

#### 2.2.1 Peters’ suppressive test

The use of *P. berghei* (a species that causes murine malaria) as a development model for analyzing almost all aspects of transmission makes it the main parasite used for the experimental study of malaria. The pattern of infection by *P. berghei* ANKA consists of a neurological syndrome that occurs 6–14 days after infection and with a mortality rate of approximately 90% (Simwela and Waters, 2022). Neurological manifestations include paraplegia, ataxia, and seizures. The remaining 10% of infected animals die within 3–4 weeks, with severe anemia, hyperparasitemia and no neurological signs (Lou et al., 2001; Simwela and Waters, 2022). Taken together, the characteristics of signs and symptoms described are similar to those exhibited in human cases of severe malaria.

The *P. berghei* ANKA strain, was donated by Instituto Evandro Chagas - Ananindeua - PA, Brazil, was replicated three times in BALB-c mice (aged between 5 and 6 weeks) and the inoculum was prepared with 1 × 10^6^ parasitized red blood cells per 0.2 mL of complete medium (RPMI 1640% and 5% fetal bovine serum), administered intraperitoneally to the mice. The animals were randomly divided into 8 groups with 10 animals each (Sham, CN, and CP groups) and 10 per subgroup (EEEp, FDMEp, isoeleutherin, eleutherin, and eleutherol), treated orally for 4 days as follows:

The animals were divided by simple randomization into 8 large groups, with only those treated with the extract, fraction and isolated substances being subsequently divided into subgroups, totaling a total of 16.

Sham control group (Sham; N = 10 animals): Sham animals were inoculated with uninfected erythocytes and received 0.9% physiological saline solution; 1 mL/100 g body weight orally for 4 days.

Negative control group (NC; N = 10 animals): The animals were inoculated with erythrocytes infected with *P. berghei* and received 0.9% physiological saline solution; 1 mL/100 g body weight orally for 4 days.

Chloroquine positive control group (N = 10 animals): The animals were inoculated with *P. berghei* in the same way as the CN group and treated with chloroquine 30 mg/kg body weight orally for 4 days.

Group treated with ethanolic extract (EEEp; N = 30 total, with n = 10 animals for the three dose subgroups 50, 100, and 200 mg/kg): The animals were inoculated with P. *berghei* in the same way as the CN group and treated orally for 4 days with EEEp, in subgroup 1 with a dose of 50 mg/kg, in subgroup 2 with a dose of 100 mg/kg and in subgroup 3 with a dose of 200 mg/kg of animal weight.

Group treated with the dichloromethane fraction (FDMEp; N = 30 total, with n = 10 animals for the three dose subgroups 50, 100, and 200 mg/kg): The animals were inoculated with *P. berghei* in the same way as the group CN and treated orally for 4 days with FDMEp, in subgroup 1 with a dose of 50 mg/kg, in subgroup 2 with a dose of 100 mg/kg and in subgroup 3 with a dose of 200 mg/kg of weight animal.

Group treated with isoeleuterin (N = 30 total, with n = 10 animals for the three dose subgroups 15, 30, and 45 mg/kg): The animals were inoculated with *P. berghei* in the same way as the CN group and treated via orally for 4 days with isoeleuterine, in subgroup 1 with a dose of 15 mg/kg, in subgroup 2 with a dose of 30 mg/kg and in subgroup 3 with a dose of 45 mg/kg of animal weight.

Group treated with eleuterin (N = 20 total, with n = 10 animals for the two dose subgroups 15 and 45 mg/kg): The animals were inoculated with *P. berghei* in the same way as the CN group and treated orally for 4 days with eleuterine, in subgroup 1 with a dose of 15 mg/kg and in subgroup 2 with a dose of 45 mg/kg of animal weight. It was not possible to test the 30 mg dose due to the low yield of the isolated substance.

Group treated with eleutherol (N = 20 total, with n = 10 animals for the two dose subgroups 15 and 45 mg/kg): The animals were inoculated with *P. berghei* in the same way as the CN group and treated orally for 4 days with eleutherol, in subgroup 1 with a dose of 15 mg/kg and in subgroup 2 with a dose of 45 mg/kg of animal weight. It was not possible to test the 30 mg dose due to the low yield of the isolated substance.

At the end of the study period, surviving animals were intraperitoneally anesthetized with ketamine (1.5 μL/g body weight) and xylazine (0.5 μL/g body weight) and euthanized by exsanguination (hypovolemia) to obtain whole blood samples by cardiac puncture. Whole blood was collected with heparin and centrifuged at 2,500 rpm for 15 min and stored in conical tubes in a freezer at −70°C for subsequent immunological analysis. All sacrificed animals were placed in biological material plastic bags, then frozen until collection and disposal by the specialized company contracted by UFPA, according to the Health Waste Management Plan (PGRS) of the institution.

After the infection and treatment period, the animals were monitored daily for 30 days, recording the number of deaths that occurred during the experiment and to which group the animal belonged. To verify the parasitemia of the mice, blood smears were performed on the 5^th^ and seventh days after parasite inoculation, obtained from the animals by caudal vein puncture. The blood smear on a microscope slide, once dried at room temperature, was fixed with methanol for 2 min and stained with Giemsa reagent for 10 min, followed by washing the stained slide with running water. After drying the slides, parasitized red blood cells were counted under an optical microscope at ×100 magnification using immersion oil. A reduction of 30% or more in *P. berghei* growth was established as the criterion for *in vivo* antimalarial activity of the tested compounds (Table 1) (Carvalho and Krettli, 1991; Andrade-Neto et al., 2003; Andrade- Neto et al., 2004).

**TABLE 1:** Number of red blood cells to be counted from an initial estimate of parasitemia.

% parasitemia	Red blood cells to be counted
0	10.000
<6	5.000
6–10	2.000
11 a 20	1.000
>20	300

% = percentage. Source: Carvalho and Krettli, 1991.

Female mice were used, as the hormonal presence can influence the immune response, especially in infectious diseases. Furthermore, the presence of expression of the double dose of the X chromosome in females would be another factor that could influence. Regarding treatments, pathogenesis studies, involving pharmacokinetics and pharmacodynamics in trial phases, use females as the greatest differences in susceptibility to malaria are focused on females (Fish, 2008; Van Lunzen and Altfeld, 2014).

#### 2.2.2 Determination of IL-10, IFN-γ, and nitrites and nitrates levels

The concentrations of IL-10 and IFN-γ cytokines were evaluated in the plasma of animals according to the study period. Cytokines were quantified using commercial ELISA kits according to the manufacturers’ instructions. All quantified cytokines operate on the same principle, where anti-cytokine capture antibodies are sensitized in 96-well plates, and depending on the cytokine present in the samples, they specifically bind to the antibody. The colorimetric reaction occurs after the addition of the second biotin-avidin-peroxidase-labeled anti-cytokine antibody in the presence of tetramethylbenzidine (TMB) substrate. After adding TMB, the reaction is stopped by adding 1 M sulfuric acid. Cytokine quantification was detected by colorimetry in a microplate reader at a wavelength of 492 nm, and the concentration for each sample was calculated from the corresponding standard curve in pg/mg of protein.

The determination of nitric oxide metabolites was performed using the Nitrate/Nitrite Colorimetric Assay Kit (Cayman Chemical, Cat # 780). The measurement of nitric oxide metabolites was carried out using a colorimetric method, using an ELISA plate as a support, in which the use of nitrate reductase promoted the conversion of nitrate to nitrite. Next, the Griess reagent converted the nitrite into an azo compound with an intense purple color, with absorbance between 540 and 550 nm. Additionally, with the aim of minimizing the interference produced in the Griess reagent by NADPH (NOS enzyme cofactor), small amounts of NADPH were conjugated with a reducing catalytic system, preventing the oxidation of NADPH to NADP+. The absorbance results were read on a multiplate reader at 540 nm (PerkinElmer, Victor X3).

#### 2.2.3 Statistical analysis

Statistical analysis was conducted using GraphPad Prism 5. For each analyzed parameter, an assessment of possible outliers was performed using the interquartile range, with any outliers excluded from the statistical calculations. Homoscedasticity of dispersion was evaluated for each parameter using Analysis of Variance (ANOVA). After identifying significant differences, these were compared between groups using the Tukey *post hoc* test. A significance level of 5% (*p* ≤ 0.05) was considered for all tests. Pearson correlation test was performed to assess potential correlations between parameters, considering the pairing of survival or parasitemia with IL10, TNF-α, or NO, with a significance level of 5% (*p* < 0.05).

#### 2.2.4 Metabolism simulation and molecular docking

2D structures were drawn using MarvinSketch™ and optimized in Avogadro™ to their lowest energy and highest stability form, applying the MMFF94 force field. Subsequently, the molecules underwent metabolism prediction via the XenoSite™ web server (https://xenosite.org/), which utilizes the Site of Metabolism (SOMs) system to evaluate the metabolism of cytochrome P450 isoforms using a machine learning approach with neurais networks (Matlock, Hughes, Swamidass, 2015).

For molecular docking, the enzymes used, such as Lactate Dehydrogenase (1OC4), were obtained from the public domain RCSB PDB (https://www.rcsb.org/) and optimized using the APBS server (https://server.poissonboltzmann.org/), where charges and polar hydrogens were added, and water and co-crystallized components with the enzymes were removed. The DockThor™ web server (https://dockthor.lncc.br/v2/) was utilized for docking, allowing both macromolecules and ligands to freely rotate. The docking study was carried out centered from the NAD using a grid-box size of X 40, Y 40, and Z 40 in virtual screening mode (number of evaluations 500,000), docking was performed three times, and an average Root Mean Square Deviation was obtained. Results were observed via Biovia Studio.

## 3 Results

### 3.1 Antimalarial activity and changes in immune response

In the Peters’ suppressive test, parasitemia and survival time of the animals should be evaluated. Regarding the parasitemia of the animals treated with EEEp, FDMEp, isoeleutherin, and eleutherol, a more pronounced reduction in parasitemia was observed on the 5^th^ day. However, in the positive control, chloroquine, the greatest reduction was observed on the 8th day. Eleutherin, *in vivo*, did not reduce parasitemia at any of the doses and times evaluated (Table 2).

**TABLE 2:** Effect of treatments with *E. plicata* extract and fraction on parasitemia of mice infected with *Plasmodium berghei*.

Groups (mg/kg)	Parasitaemia (%)		Reduction parasitemia (%)	
	5°day	8°day	5°day	8°day
EEEp 50	1.93	7.82	24.31	3.45
EEEp 100	1.72	7.43	32.54	8.27
EEEp 200	1.38	6.63	45.88	18.14
FDMEp 50	1.88	7.6	26.27	6.17
FDMEp 100	1.75	7.48	31.37	7.65
FDMEp 200	0.9	5.41	64.7	33.2
Isoeleutherin 15	2.33	8.34	8.62	-
Isoeleutherin 30	2.83	8.26	10.9	-
Isoeleutherin 40	2.08	7.76	18.43	4.19
Eleutherin 15	2.62	8.4	-	-
Eleutherin 45	2.81	8.23	-	-
Eleutherol 15	1.71	7.48	32.94	7.65
Eleutherol 45	2.12	7.89	16.86	2.59
Positive Control	0.5	0.03	80.4	99.62
Negative Control	2.55	8.1	-	-

Legend: EEEp, ethanolic extract of *E. plicata*; FDMEp, dichloromethane fraction of *E. plicata*; Positive control - chloroquine 30 mg/kg; Negative control - untreated. (%) Mean parasitemia of the animals. The reduction in parasitemia was calculated relative to the mean parasitemia of the negative control group.

The study evaluates the total parasite load of infected red blood cells, including trophozoites and schizonts. Parasitized red blood cells were counted under an optical microscope at ×100 magnification using immersion oil, and the estimate of the number of parasitized red blood cells was decisive for the number of fields counted, as shown in Table 1 below. The percentage ratio between the number of parasitized red blood cells and the total number of counted red blood cells was considered as the degree of parasitemia.

Another important aspect evaluated was the interference of the dose used in reducing parasitemia, where EEEp, FDMEp, and isoeleutherin showed the greatest reductions in parasitemia in animals treated with the highest doses. In the case of eleutherol, a greater reduction in parasitemia was observed in animals treated with the lowest dose (Table 2).

**FIGURE 1:**
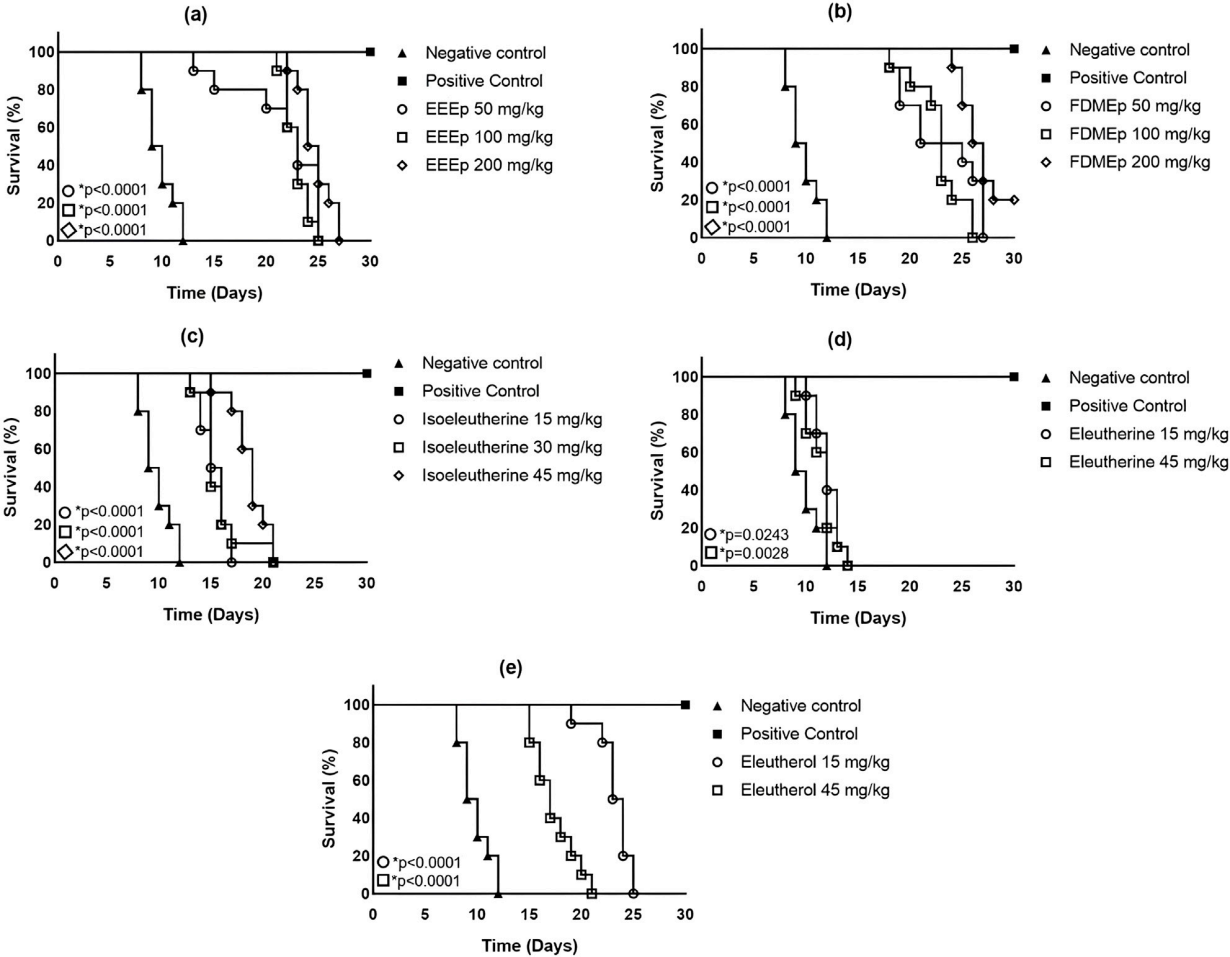
Survival of animals treated with extract, fraction, and compounds obtained from *E. plicata.* Legend. **(A)** EEEp - ethanolic extract of *E. plicata*, **(B)** FDMEp - dichloromethane fraction of *E. plicata*, **(C)** Isoeleutherin, **(D)** Eleutherin, and **(E)** Eleutherol. *p < 0.05 (compared to the negative control).

Regarding survival time, it was observed that untreated infected animals (negative control) survived for a maximum of 12 days. While in the group treated with chloroquine (positive control), there were no deaths recorded in 30 days of the experiment. In animals treated with EEEp (maximum survival time ≥20 days), FDMEp (maximum survival time ≥25 days), isoeleutherin (maximum survival time >15 days), and eleutherol (maximum survival time ≥20 days), there was an increase in survival time compared to the negative control. Regarding eleutherin, there was a slight increase in the survival time of animals treated with this naphthoquinone (Figure 1).

**FIGURE 2:**
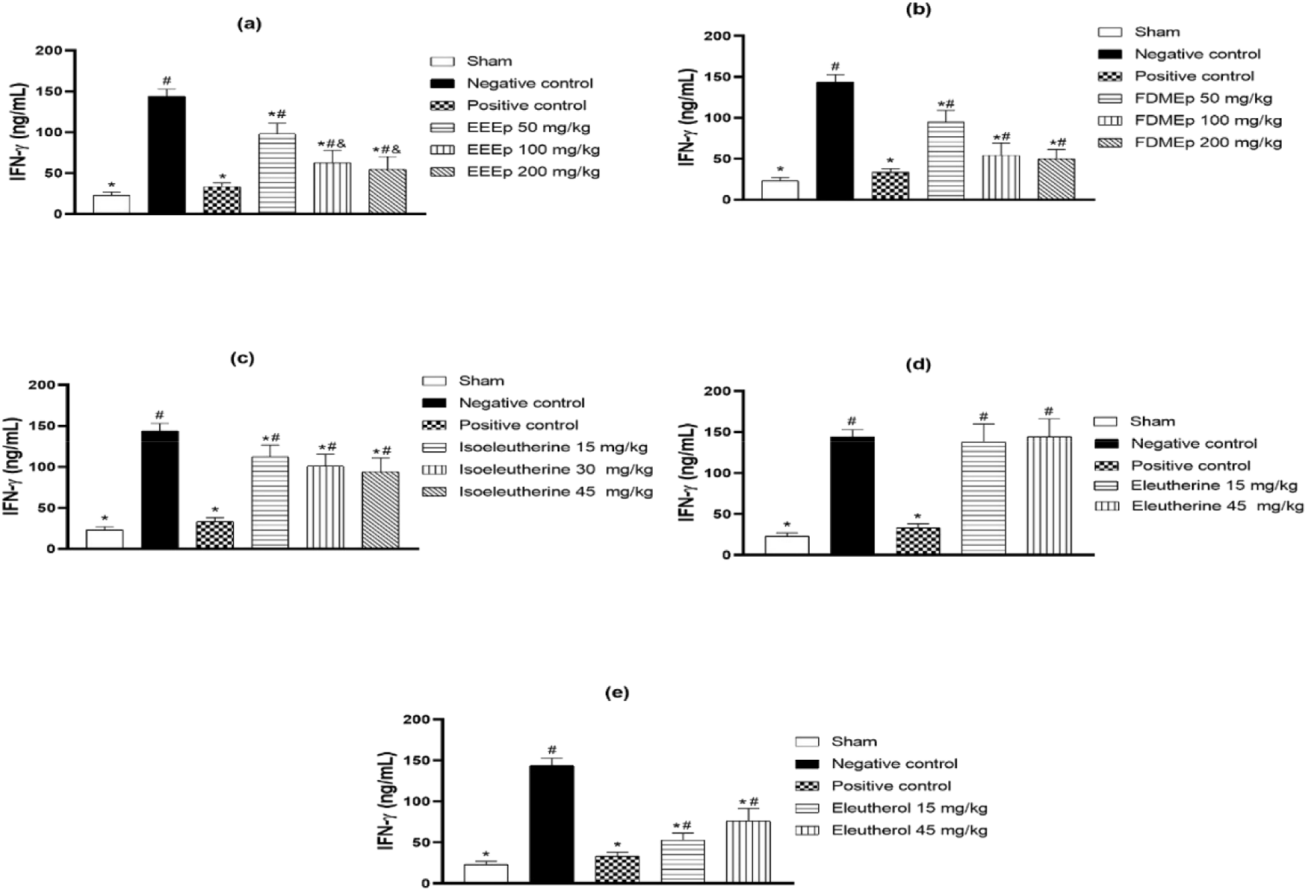
Levels of IFN-γ in animals treated with extract, fraction, and compounds obtained from *E. plicata*. Legend. **(A)** EEEp - ethanolic extract of *E. plicata*, **(B)** FDMEp - dichloromethane fraction of *E. plicata*, **(C)** Isoeleutherin, **(D)** Eleutherin, and **(E)** Eleutherol. *p < 0.05 (compared to the negative control); #*p* < 0.05 (compared to the positive control); and *p* < 0.05 (comparison between treatment doses).

The impact of treatment on IFN-γ levels in animals treated with *E. plicata* and derivatives was evaluated. Similarly to parasitemia, the greatest reduction in IFN-γ occurred in animals treated with the highest doses of EEEp, FDMEp, and isoleutherin. Also, similarly to the reduction in parasitemia, animals treated with the lowest dose of eleutherol showed a greater reduction in IFN-γ. Eleutherin did not interfere with IFN-γ levels, and in the chloroquine-treated group (positive control), there was a significant reduction in IFN-γ (Figure 2).

**FIGURE 3:**
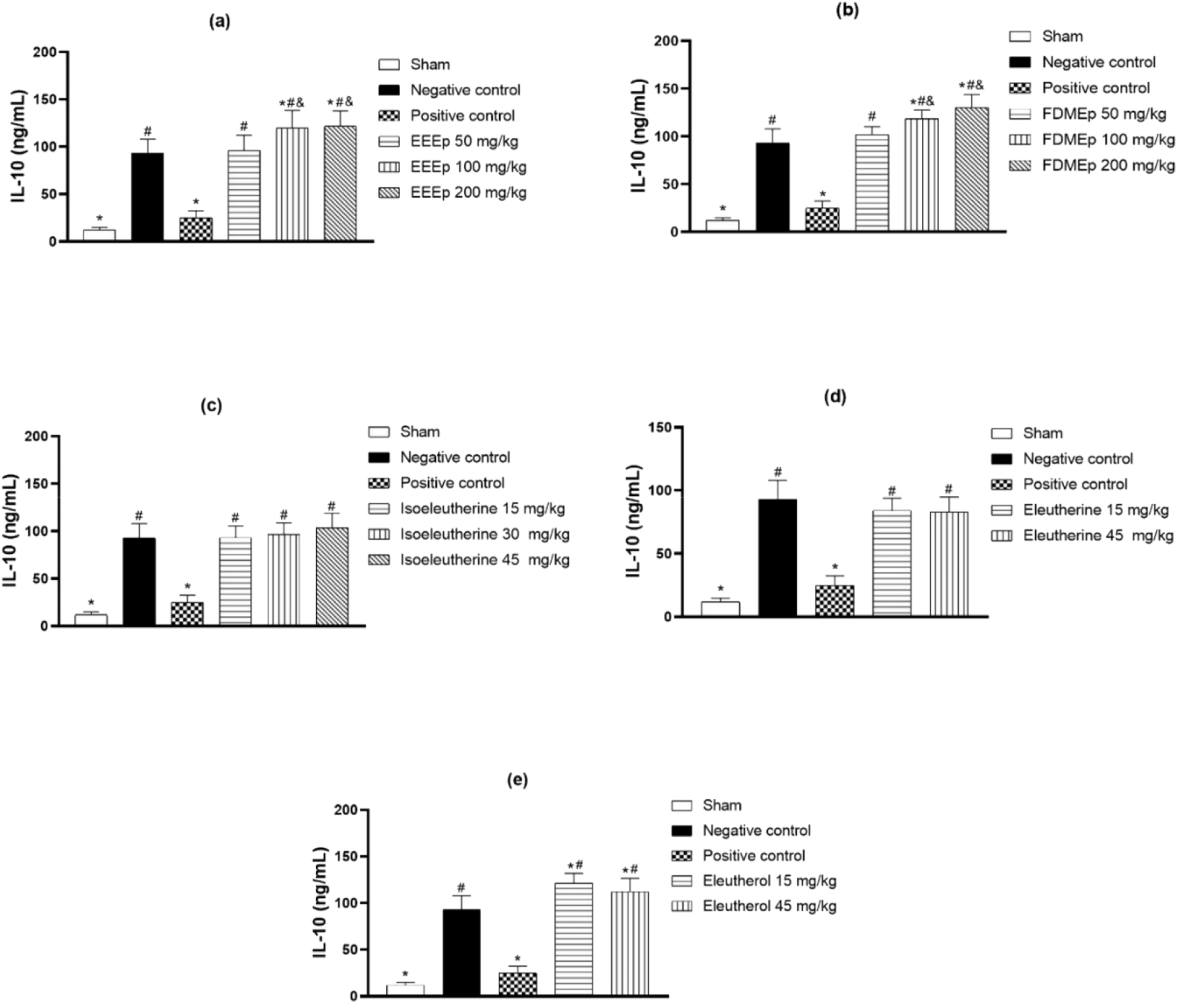
Levels of IL-10 in animals treated with extract, fraction, and compounds obtained from *E. plicata.* Legend. **(A)** EEEp - ethanolic extract of *E. plicata*, **(B)** FDMEp - dichloromethane fraction of *E. plicata*, **(C)** Isoeleutherin; **(D)** Eleutherin; and **(E)** Eleutherol. *p < 0.05 (compared to the negative control); #*p* < 0.05 (compared to the positive control); &*p* < 0.05 (comparison between treatment doses).

In the case of IL-10 dosage, there was a greater elevation in its levels in the groups treated with the highest doses of EEEp and FDMEp. In animals treated with isoleutherin, eleutherin, and eleutherol, there was no clear relationship between the dose and IL-10 levels. Unlike *E. plicata* and derivatives, where elevated levels of IL-10 were observed, in the group treated with chloroquine, there was a reduction in IL-10 levels (Figure 3).

**FIGURE 4:**
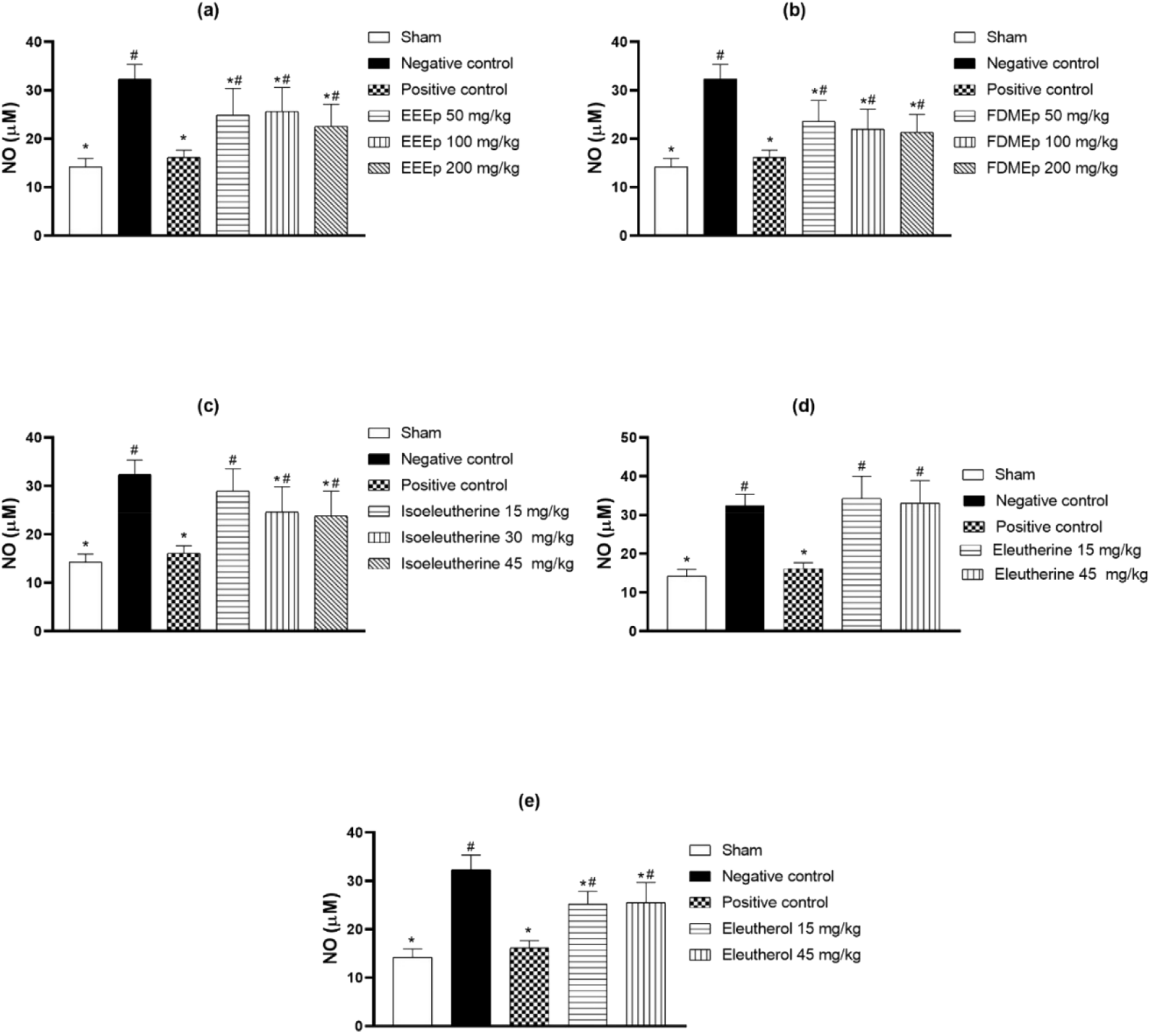
Levels of nitrate/nitrite in animals treated with extract, fraction, and compounds obtained from *E. plicata.* Legend. **(A)** EEEp - ethanolic extract of *E. plicata*, **(B)** FDMEp - dichloromethane fraction of *E. plicata*, **(C)** Isoeleutherin, **(D)** Eleutherin, and **(E)** Eleutherol. *p < 0.05 (compared to the negative control); #*p* < 0.05 (compared to the positive control); &*p* < 0.05 (comparison between treatment doses).

Regarding NO, the highest levels were observed in the negative control group, and in the positive control, there was a significant reduction in these levels. However, in the groups treated with EEEp, FDMEp, isoleutherin, and eleutherol, there was no relationship between the doses used and the reduction of NO. In the case of eleutherin, the levels of NO were similar to the negative control (Figure 4).

The reduction in parasitemia appears to have a direct relationship with the survival time of animals infected with *P. berghei*; that is, the greater the reduction in parasitemia, the longer the life expectancy. Thus, the highest survival rates and reductions in parasitemia were observed for FDMEp and chloroquine, and the lowest survival rate was for eleutherin, which has no antiparasitic effect.

When relating IFN-γ levels to parasitemia, it is also observed that the greater the reduction in parasitemia, the greater the reduction in IFN-γ. When there was no antiparasitic effect, as in the case of eleutherin, no reduction in IFN-γ was observed. Similar relationships were also observed for IL10 and NO. However, correlation studies were conducted between parasitemia and IFN-γ, IL10, and NO levels, revealing a positive correlation between IFN-γ and eleutherin, where no antiparasitic effect was observed. Regarding correlation studies between levels of IFN-γ, IL-10, and NO, a correlation was observed between IFN-γ and NO with isoleutherin. Correlations were also observed between IL10 and eleutherin and FDMEp.

### 3.2 Simulation of metabolism and molecular docking

**FIGURE 5:**
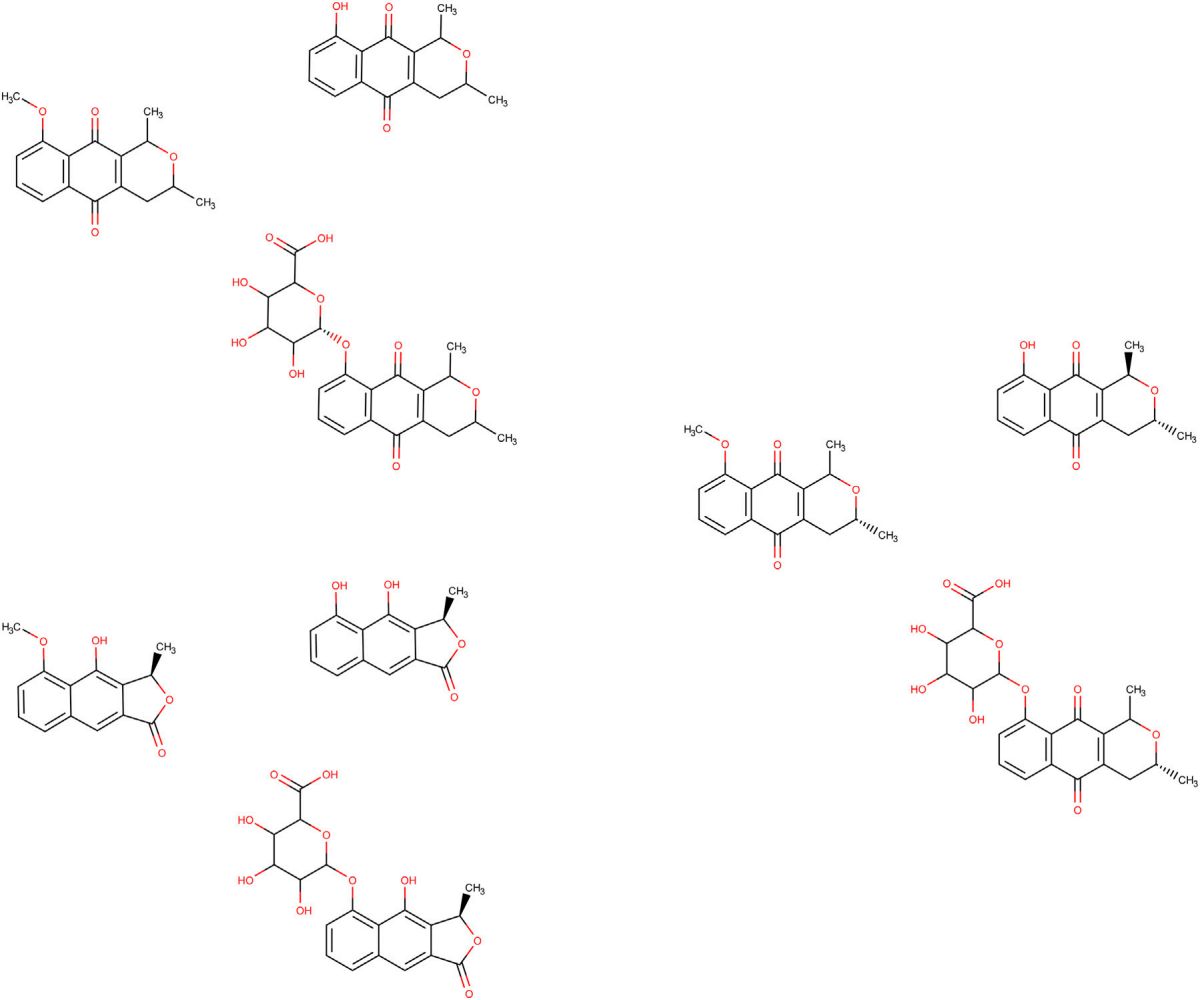
Metabolism products predicted by XenoSite™ Legend. **(A)** Eleutherin, **(A1)**: Demethylated eleutherin and **(A2)**: eleutherin glucuronide; **(B)** Eleutherol, **(B1)**: Demethylated eleutherol and **(B2)**: Eleutherol glucuronide; **(C)**. Isoeleutherin, **(C1)**: Demethylated isoeleutherin and **(C2)**: Isoeleutherin glucuronide.

According to the XenoSite™ server, eleutherol, eleutherin, and isoeleutherin each exhibited two metabolisms, one of the first phase (CYP 2C9 or 2C19) and another of conjugation with uridine diphosphate glucuronosyltransferases (UGT; Figure 5), both with a probability greater than 0.7. The methyl attached to the hydrogen via first-phase mechanism in both was removed forming a hydroxyl, generating the products named demethylated eleutherol, demethylated eleutherin, and demethylated isoeleutherin, similarly, in a new reaction, from the conjugation with UGT, the same methyl was removed and the glucuronic acid was added forming another three products named eleutherol glucuronide, eleutherin glucuronide, and isoeleutherin glucuronide. All these ligands were subjected to molecular docking on the three targets studied.

Table 3 summarizes the affinity (Kcal/mol) obtained by all compounds under study, in addition to the number of bonds made with the macromolecules and the involved amino acid residues. During docking, it was observed that all compounds demonstrated high affinity for the macromolecules, showing negative values in their scores.

**TABLE 3:** Affinity of naphthoquinones and their metabolites with Lactate dehydrogenase (1OC4).

Ligands	Affinity (Kcal/mol)	Number of bonds/interactions with amino acids	Root mean square deviation (RMSD)
1OC4			
Eleutherin	−8.18	6; His^473^, Leu^584^, Thr^186^	1.8 Å
Eleutherin demethylated	−6.45	6; Arg^468^, Arg^164^, Lys^548^, Glu^540^	0.9 Å
Eleutherin glucuronide	−6.87	11; Arg^468^, Arg^164^, Arg^240^, Lys^25^, Lys^244^, Glu^236^	0.7 Å
Isoeletherin	−6.35	10; Arg^468^, Ala^533^, Ile^536^, Lys^329^, Lys^548^, Glu^537^, Glu^540^	2.1 Å
Isoeletherin demethylated	−6.34	5; Arg^468^, Arg^164^, Lys^548^, Glu^540^	0.7 Å
Isoeletherin glucuronide	−6.82	8; Arg^240,^ Arg^468^, Leu^242^, Lys^25^, Glu^236^	0.2 Å
Eleutherol	−7.72	7; Arg^487^, Glu^190^, Val^187^, Tyr^488^	1.7 Å
Eleutherol demethylated	−6.82	4; Arg^547^, Asp^545^, Tyr^153^	1.8 Å
Eleutherol glucuronide	−8.43	8; Ala^226^, Ala^229^, Arg^150^, Lys^152^, Ser^149^, Pro^163^	0.8 Å

## 4 Discussion

The initial expectations of this study were that the fractionation of EEEp would contribute to the anti-malarial activity and that the naphthoquinones eleutherin and isoeleutherin would be responsible for the anti-malarial activity, while eleuterol would not present anti-malarial potential (Vale et al., 2020). However, this study showed that eleutherin did not reduce parasitemia, isoeleutherin caused a slight reduction, and eleuterol was the most active compound. These discrepancies in the results suggest that the compound responsible for the anti-parasitic activity of eleuterol must be related to its glucuronide.

To understand this process, lactate dehydrogenase (LDH) enzyme was the target focused on *in silico* study. As observed, eleuterol glucuronide showed higher affinity with this target. LDH is an important enzyme in the parasite’s survival, as it participates in glycolysis converting pyruvate into lactate. During parasitemia, upon degrading the erythrocyte, ferriprotoporphyrin IX (hemozoin) becomes toxic to the parasite, which, using the polymerization process, converts it into an inert pigment, hemozoin. Thus, by preventing the formation of hemozoin by binding to LDH sites, eleuterol glucuronide may induce parasite death through the toxic effect of hemozoin (Pena-Coutinho et al., 2011). LDH is also an enzyme that participates in the metabolism in mammals, which succincts further studies regarding the selectivity of compounds between mammals and parasites, however, studies have demonstrated that there is a certain selectivity for the hyperactivation of the pathway by *Plasmodium* (Kayamba et al., 2021; He et al., 2022).

A previous study demonstrated that despite the good intestinal permeability of isoeleutherin, eleutherin and eleutherol, the bioavailability of these compounds is low (5.38%, 4.64% and 2.47%, respectively) (Guo et al., 2022). Thus, it can be said that eleutherin may not have antiparasitic activity in its non-metabolized form, which may explain the differences between the *in vitro* and *in vivo* results. Furthermore, the metabolism of eleutherol may be responsible for the antiparasitic effect, since it alone presented significant results and the association of the metabolite of these compounds with others with anti-inflammatory activities contributes to the effects found for the FDMEp. An important fact is that FDMEp seems to be the most promising as an *in vivo* anti-malarial, with a reduction in parasitemia observed on the 5th and 8th evaluation days. A previous study demonstrated that isoeleutherin and eleuterol are present in FDMEp (Castro et al., 2021), suggesting that the pharmacological effect may result from the synergy between isoeleutherin and the eleuterol metabolite.

An assessment of the genotoxicity of *E. plicata* using the comet test demonstrated that the fractionation of EEEp led to obtaining FDMEp with greater genotoxic potential than EEEp. Isoeleutherin was even more genotoxic than FDMEp and EEEp (Gomes et al., 2021). Using the micronucleus model, the genotoxicity of the same samples was evaluated, and it was observed that the highest micronucleus frequency was found for FDMEp (Albuquerque et al., 2024). However, in the acute oral assay, animals treated with 2000 mg/kg of EEEp, FDMEp, and isoeleutherin showed no signs of toxicity, and there were no deaths, similar results were found in the subacute oral toxicity assay for EEEp (1000 mg/kg) and FDMEp (1000 mg/kg) (Gomes et al., 2021). Due to the *in vitro* results obtained with FDMEp, it is important to conduct *in vitro* studies to evaluate the genotoxic potential. As previously discussed, *in vivo*, compound metabolism occurs, and metabolites may present a different activity and genotoxicity profile.

In general, the survival time of Swiss mice infected with *P. berghei* is 20 days, but with *P. berghei* ANKA with high parasitemia, this time reduces to 9 days (Pereira, 2016). In this study, BALB-c mice were used, and thus, the survival time of untreated and infected animals was approximately 12 days. The relationship between survival time and parasitemia was observed, where animals treated with EEEp, FDMEp, and eleuterol had the highest reductions in parasitemia and the longest survival time.

It is known that the immune response occurs in the exoerythrocytic phase, with the production of pro-inflammatory cytokines such as IFN-γ and TNF-α, which try to eliminate the parasite through the NO pathway and the production of interleukins IL1, IL6, and IL8, as well as through CD8^+^ and CD4^+^ T cells acting through cytolytic action or producing pro-inflammatory cytokines. The immune response continues in the erythrocytic phase, being mediated by macrophages and CD4^+^ helper T cells of the Th1 and Th2 subtypes, with production of IFN-y, oxygen, and nitrogen radicals, and cytokine production (Queiroz, Teixeira, Teixeira, 2008). Due to the importance of the immune response to malaria, this study evaluated the activity of EEEp, FDMEp, and compounds on the important mediator IFN-γ and observed that EEEp, FDMEp, and eleuterol significantly reduced IFN-γ levels.

IFN-γ is involved in the regulation of the immune and inflammatory response (Farrington et al., 2017; Kumar et al., 2019), and its reduction by EEEp, FDMEp, and eleuterol may signal that, in addition to the anti-parasitic effect, these samples may reduce the inflammatory process caused by *Plasmodium* infection.

Patients with malaria present reduced levels of IL-10, an anti-inflammatory interleukin, which may be related to thermal hyperalgesia occurring in mice infected with *P. berghei*, especially in the late stages of infection. Also, the reduction in latency is related to the increase in parasitemia in infected mice and low serum levels of IL10 (Nakamae et al., 2019). In this study, it was observed that, mainly, EEEp, FDMEp, and eleuterol increase IL10 levels, which may interfere with the hyperalgesia process.

It is known that *falciparum* malaria can worsen, leading to cerebral malaria, which is related to hyperactivity of the immune response (Gazzinelli et al., 2014; Dunst et al., 2017). The presence of infected erythrocytes and cellular material resulting from the rupture of these parasitized cells in circulation activates the host’s immune response, and the innate immune system stimulates macrophages to secrete pro-inflammatory cytokines. TNF-α and INF-γ lead to the activation of endothelial cells, which start to express adhesion molecules, facilitating the sequestration of parasitized erythrocytes and triggering local inflammation (Shabani et al., 2017).

Cerebral microvascular obstruction leads to endothelial dysfunction and consequent ischemia (Desruisseaux et al., 2010; Carvalho et al., 2014; Eisenhut, 2015). NO promotes vasodilation, diffusing from the endothelium to adjacent vascular smooth muscle, activating soluble guanylate cyclase to generate cyclic guanosine monophosphate, which promotes relaxation of smooth muscle cells (Kampoli et al., 2012; Pradhan et al., 2018). In cerebral malaria, there is a low availability of NO and low plasma concentrations of L-arginine, a substrate for NO synthesis (Lopansri et al., 2003), and hypoargininemia has been strongly associated with mortality (Alkaitis et al., 2016; Gupta et al., 2017). In the present study, an elevation of NO was observed in infected animals, and this elevation was partially reduced by EEEp, FDMEp, and eleuterol.

A study has shown that infection by *P. vivax* in humans induces a more intense inflammatory immune response than that induced by *P. falciparum*. To understand the possible mechanisms involved, biomarkers were sought, such as microparticles (MP) resulting from cell activation and/or apoptosis and circulating nucleic acids (CNA) associated with *P. vivax* infection. Clinically, it was possible to demonstrate that MPs from different origins were increased in *P. vivax* malaria, with platelet-derived MPs being predominant. Plasma CNA levels were increased, and these levels were associated with the clinical spectrum of the disease. There was a correlation between CNA levels and thrombocytopenia. MP and CNA levels in plasma returned to baseline values after antimalarial treatment. The association between genetic polymorphisms of cytokines (MIF, TNF-α, IL-10, and TGF-β) and platelet glycoproteins (GPIa/IIa and GPIIb/IIIa) with clinical and hematological complications of *P. vivax* infection was evaluated. The results showed associations between polymorphisms of the GPIa/IIa, TNF-α, MIF, and IL-10 genes and clinical and hematological alterations (Campos, 2011).

Dialogically, we have atovaquone, also from the naphthoquinones class, possessing the same mechanism found by Vale et al. (2020) for eleutherin and isoeleutherin, acting on the parasite’s DNA acting on its replication, it interferes with the mitochondrial electron transport chain, inhibiting cytochrome bc_*1*_, leading to a decrease in parasitemia (Cottrell et al., 2014; Gomes et al., 2021). Therefore, what we observed in this study may be correlated to the mechanisms already described for naphthoquinones, bringing news paths demonstrate the great potential of the species against malaria.

Analyzing the results obtained so far, it seems that FDMEp and eleuterol, to a lesser extent EEEp, are promising for the treatment of the erythrocytic forms of *P. vivax*, potentially acting as rapid blood schizonticides, as well as altering the immune response caused by the parasite. Unfortunately, there are still no experimental models that allow evaluating the activity of EEEp, FDMEp, and eleuterol in *P. vivax* and the changes in the immune response.

## 5 Conclusion

The results demonstrated the antimalarial potential of FDMEp and eleuterol, reducing parasitemia, increasing survival time, reducing INF-γ, and elevating IL-10. EEEp and isoeleutherin also reduced parasitemia and INF-γ to a lesser extent, with an increase in IL-10. Eleutherin showed no antimalarial activity at the doses evaluated. Unlike *in vitro* studies where the naphthoquinones isoeleutherin and eleutherin were responsible for the antiplasmodial activity, *in vivo*, eleuterol showed greater antimalarial potential. These results suggest that the eleuterol glucuronide metabolite is likely responsible for the antimalarial activity.

## Data Availability

The original contributions presented in the study are included in the article/supplementary material, further inquiries can be directed to the corresponding authors.
